# Comparison of Different Dietary Indices as Predictors of Inflammation, Oxidative Stress and Intestinal Microbiota in Middle-Aged and Elderly Subjects

**DOI:** 10.3390/nu12123828

**Published:** 2020-12-15

**Authors:** Sergio Ruiz-Saavedra, Nuria Salazar, Ana Suárez, Clara G. de los Reyes-Gavilán, Miguel Gueimonde, Sonia González

**Affiliations:** 1 Department of Microbiology and Biochemistry of Dairy Products, Instituto de Productos Lácteos de Asturias (IPLA-CSIC), 33300 Villaviciosa, Asturias, Spain; sergioruizsa3@gmail.com (S.R.-S.); nuriasg@ipla.csic.es (N.S.); greyes_gavilan@ipla.csic.es (C.G.d.l.R.-G.); mgueimonde@ipla.csic.es (M.G.); 2 Department of Functional Biology, University of Oviedo, 33006 Oviedo, Spain; anasua@uniovi.es; 3 Metabolism Area, Instituto de Investigación Sanitaria del Principado de Asturias (ISPA), 33011 Oviedo, Spain

**Keywords:** dietary patterns, Mediterranean diet, dietary indices, microbiota, elderly

## Abstract

During the last decades the gut microbiota has been identified as a key mediator in the diet-health interaction. However, our understanding on the impact of general diet upon microbiota is still limited. Dietary indices represent an essential approach for addressing the link between diet and health from a holistic point of view. Our aim was to test the predictive potential of seven dietary ratings on biomarkers of inflammation, oxidative stress and on the composition and metabolic activity of the intestinal microbiota. A cross-sectional descriptive study was conducted on a sample of 73 subjects aged >50 years with non-declared pathologies. Dietary inflammatory index (DII), Empirical Dietary Inflammatory Index (EDII), Healthy Eating Index (HEI), Alternative Healthy Eating Index (AHEI), Mediterranean adapted Diet Quality Index-International (DQI-I), Modified Mediterranean Diet Score (MMDS) and relative Mediterranean Diet Score (rMED) were calculated based on a Food Frequency Questionnaire. Major phylogenetic types of the intestinal microbiota were determined by real time polymerase chain reaction (qPCR) and fecal short chain fatty acids (SCFAs) by gas chromatography. While DII, HEI, DQI-I and MMDS were identified as predictors of *Faecalibacterium prausnitzii* levels, AHEI and MMDS were negatively associated with *Lactobacillus* group. HEI, AHEI and MMDS were positively associated with fecal SCFAs. In addition, DII and EDII explained lipoperoxidation level and Mediterranean scores the serum IL-8 concentrations. The lower detection of IL-8 in individuals with higher scores on Mediterranean indices may be partially explained by the increased levels of the anti-inflammatory bacterium *F. prausnitzii* in such individuals.

## 1. Introduction

The empirical relationship between diet and health has been recognized since the time of Hippocrates (400 BC). During the last decades, solid scientific evidence has accumulated on the protective role of certain foods, such as fruits and vegetables, on the risk of suffering non-transmissible diseases [1,2]. However, understanding the net impact of diet on health is more complex than studying isolated components. Humans do not consume single foods but a wide variety of combinations of foods forming the so-called dietary pattern. Therefore, from a physiological point of view, the analysis of the eating habits, considering the interactions between different foods and their components, is of paramount interest [3,4]. In this context, dietary indices have been developed as a useful tool for categorizing dietary practices in different populations, encouraging the comparison among different studies [5,6]. The currently available dietary indices could be clustered into three main categories: the inflammatory ones [7,8], those quantifying the adherence to dietary guidelines [9,10] and those evaluating the degree of adaptation to the Mediterranean dietary pattern [11]. Both, the number and type of components included in each index is different, depending on the purpose for which they have been created and the dietary habits of the population for which they have been designed. While inflammatory ratings, such as the Dietary Inflammatory Index (DII) [7] or the Empirical Dietary Inflammatory Index (EDII) [8], have proven to be useful in the prediction of inflammatory parameters such as C-reactive protein (CRP), interleukin 6 (IL-6) or adiponectin levels [12,13,14], the Healthy Eating Index (HEI) [15] or the Alternative Healthy Eating Index (AHEI) [10], based on diet quality, have been useful for assessing the risk of chronic diseases [10,16]. Among the different indicators, the Mediterranean dietary index is perhaps the one accumulating more scientific evidence about its beneficial impact on morbidity and mortality [17] through the reduction of different parameters related to oxidative stress [18,19].

Therefore, these indices represent a key tool in the assessment of the association between diet and health. Moreover, the inclusion of some novel biological parameters, such as the gut microbiota, in the study of such correlations may broaden their applicability [20,21]. In this regard, HEI and Mediterranean ratings were recently found to be associated with gut microbiota in terms of both microbial composition and diversity [22,23,24]. In more detail, MedDietScore index has been associated with higher fecal bifidobacteria: *Escherichia coli* ratio, total bacteria and short chain fatty acids (SCFAs) [25]. However, the studies in this area are still limited and there are no studies comparing the different indices for assessing the interaction between regular diet and microbiota.

In view of this evidence, the main objective of this work was to analyze the diet of a group of middle aged and elderly participants, without declared pathologies, through different dietary indices and to examine their predictive potential on parameters related to inflammation, oxidative stress and the composition and metabolic activity of the intestinal microbiota.

## 2. Materials and Methods

### 2.1. Participants

The sample included 73 volunteers recruited in Asturias (North of Spain), aged between 56 and 95 years and with a Body Mass Index (BMI) [weight (kg)/height (m^2^)] from 19.9 to 37.5 kg/m^2^. In an individual interview, volunteers were informed about the objectives of the study and an informed written consent was obtained before enrolment. Exclusion criteria were the presence of diagnosed immune or digestive related pathologies as well as consumption of corticoids, immunosuppressive drugs, monoclonal antibodies, probiotics or antibiotics in the previous month. The study was approved by the Regional Ethics Committee for Clinical Research (Servicio de Salud del Principado de Asturias nº 17/2010).

### 2.2. Nutritional Assessment

A previously validated annual semi-quantitative food frequency questionnaire (FFQ) [26,27] was used by trained personnel to assess volunteers’ regular food intake in a personal interview of approximately 1 h duration. Methodological issues about dietary assessment were published elsewhere [28]. Food composition tables of CESNID (Centro de Enseñanza Superior de Nutrición Humana y Dietética) were used to transform food consumption into energy and macronutrients intake [29]. The (poly)phenol content in foods was completed using the Phenol Explorer database [30]. Fiber components were determined using the Marlett et al. food composition tables [31]. Glucosinolates levels were obtained from McNaughton et al. [32] and isothiocyanates along with aliphatic glucosinolates food content were derived from glucosinolates levels following European Prospective Investigation into Cancer and Nutrition (EPIC) criteria [33]. Glucosinolate side chains concentrations were ascertained from International Agency for Research of Cancer (IARC) data [34]. At the time of carrying out the blood extraction, height and weight were taken by standardized protocols [35]. At the same time as the FFQ interview was conducted, a questionnaire on socio-economic factors (such as level of education or type of work) and lifestyle (physical activity, smoking and self-perception of health status) was administered.

### 2.3. Dietary Indices Calculation

A calculation of seven dietary indices was carried out, including DII, EDII, HEI, AHEI, Mediterranean adapted Diet Quality Index-International (DQI-I) [36], Modified Mediterranean Diet Score (MMDS) [37] and relative Mediterranean Diet Score (rMED) [38], as shown in Supplementary Table S1. IBM SPSS program version 24.0 (IBM SPSS, Inc., Chicago, IL, USA) was used to design a database for calculating indices scores from our FFQ data.

DII scores were calculated by evaluating 35 parameters (out of 45 possible items). Components such as eugenol, ginger, saffron, turmeric, green/black tea, isoflavones, pepper, thyme/oregano and rosemary were excluded because a lack of information about them in the FFQ recordings. First, the consumption levels of parameters were standardized by subtracting daily global consumption mean and dividing by the global standard deviation. The resulting Z-scores were then converted to percentile scores and centered by doubling and subtracting one. These centered-percentile scores were multiplied by the overall food parameter-specific inflammatory effect score to obtain the ‘food parameter-specific score’ (FPES). All FPES of an individual were summed to obtain the final DII Score. Values in our sample ranged from −4.62 to 4.45, with negative scores predicting lower inflammation and positive scores higher dietary-derived inflammation.

For EDII scores, 18 components were accounted. The number of servings consumed was calculated for each component. The resulting values were multiplied by the “Weight” of the components and divided by 1000. All weighted components were summed to obtain each EDII Score, with values ranging from −1.56 to 2.21 in our sample. The more positive the result, the more prone to higher concentrations of inflammatory biomarkers.

The HEI is an index comprised of 13 components. For each parameter, the amount of a dietary component, in g, cups or oz equivalents were calculated per 1000 kcal. These densities were scored according to recommended consumption values. For negative scoring components, considered to be consumed in small quantities due to their negative impact on health, the individuals get higher scores when the consumption values are lower than the established threshold. Then, all components were summed to obtain the HEI score. HEI was reflecting a total score from 29.49 to 77.76 in our sample, with higher values reflecting a healthier diet.

AHEI score calculations procedure was quite similar to that of HEI, with 11 components in total and values from 37.34 to 80.79 in our sample. In the same way, higher values represent healthier diets. For each component, the consumption in servings per day was calculated and scored according to AHEI-2010 criteria. All components were summed to obtain the total AHEI score of each individual.

Considered the most suitable index for international analyses, DQI-I accounts for 18 components. Here, we evaluated a Mediterranean adaptation of DQI-I. The amount consumed of each component was rated from 0 to 3, 5 or 6, depending on the component and all the points were summed to obtain DQI-I scores, whose final values were between 33 and 68 (minimum and maximum). Here, a total sum of 100 would show an individual with perfect adherence to the main four categories evaluated in this index, while a score of 0 is reflecting a diet far away from recommended dietary guidelines.

Composed of 9 components, rMED and MMDS were derived from the index originally developed by Trichopoulou et al. [11] to evaluate the degree of adherence to a traditional Mediterranean diet. rMED was calculated from components intake based on the density nutrients model. Once amounts of consumption per 1000 kcal were obtained, the sample was split in tertiles for each of the nine components. According to the tertile position for each component, individuals were rated. Summing all the ratings we obtained the rMED score, ranging from 2 to 12 in our sample. In the case of MMDS, each parameter was scored 0 or 1 according to the cut-off values of the sex specific-medians among the participants. Final MMDS scores ranged from 1 to 7 in our sample. In both indices, the highest scores are showing a higher adherence to Mediterranean diet patterns.

### 2.4. Blood Biochemical Analyses

Fasting blood samples were drawn by venipuncture and centrifuged (1000× *g*, 15 min). Plasma and serum aliquots were kept at −20 °C for later analyses. Serum glucose, serum total cholesterol, serum HDL-cholesterol, serum LDL-cholesterol and serum triglycerides were determined by using an automated biochemical autoanalyzer in an independent laboratory. Serum levels of CRP were determined by ELISA (CRP Human Instant ELISA, Ebioscience, San Diego, CA, USA) and malondialdehyde (MDA) by a colorimetric assay of lipid peroxidation (Byoxytech LPO-586 assay, Oxis International S.A., Paris, France) [39]. Serum leptin was determined by ELISA (Human Leptin ELISA Development Kit 900-K90, PeproTech Inc., Rocky Hill, NJ, USA) according to the manufacturer’s instructions. Colorimetric assay P40117 (Innoprot, Innovative Technologies in Biological Systems, S.L., Spain) was used to determine total antioxidant capacity (TAC) in serum [40]. A multiplex immunoassay (Cytometric Bead Array, CBA, BD Biosciences) by flow cytometry allowed to quantify levels of serum IL-10, Tumor Necrosis Factor-Alpha (TNF-α), IL-8, IL-17 and IL-12, while the concentration of transforming growth factor (TGF)-β was determined by ELISA (BD OptEIATM, BD Biosciences). The phagocytic capacity was quantified in a FACSCanto II Flow Cytometer (Becton Dickinson, BD Biosciences, San Diego, CA) by using the Phagotest^®^ kit (Orpegen Pharma, Heildelberg, Germany). Natural killer (NK) cell activity was determined by flow cytometry, using the NKtest^®^ kit (Orpegen Pharma).

### 2.5. Fecal Collection and Microbial Analysis

Detailed instructions about fecal samples collection were given to participants who also were provided with a sterile container. After deposition, samples were immediately frozen at −20 °C and transported to the laboratory. For analyses, samples were thawed at room temperature (24 ± 2 °C), weighed, diluted 1/10 in sterile PBS and homogenized using a LabBlender 400 Stomacher (Seward Medical, London, UK) for 4 min; the DNA was extracted using the QIAamp DNA stool mini kit (Qiagen, Hilden, Germany) following previously described procedures [41]. 7500 Fast Real-Time PCR System (Applied Biosystems, Foster City, CA, USA) and SYBR Green PCR Master Mix (Applied Biosystems) were used to achieve the quantification of bacterial populations, including the major bacterial groups present in the human gut (Supplementary Table S2). Procedure instructions were published elsewhere [41]. Fecal DNA extracts were analyzed and the mean quantity per gram of fecal wet weight was calculated for each bacterial group. One milliliter of the homogenized feces were centrifuged and supernatants were analyzed by gas chromatography to determine acetate, propionate and butyrate concentrations, as previously indicated [42]. A chromatograph 6890N (Agilent Technologies Inc., Palo Alto, CA, USA) connected to a mass spectrometry detector (MS) 5973N (Agilent Technologies) and a flame ionization detector (FID) was used for identification and quantification of SCFAs, respectively, as described previously [43].

### 2.6. Statistical Analyses

Statistical analysis was performed using the IBM SPSS program version 24.0 (IBM SPSS, Inc., Chicago, IL, USA). Mean dietary scores were analyzed by a Student t-test and Bonferroni multiple comparison according to general, socio-economic and health-related characteristics, such as smoking status, educational level, mood feeling or self-health perception among others. Similar procedure was performed to analyze mean levels of microbiological and blood variables according to age group. Goodness of fit to the normal distribution was analyzed employing the Kolmogorov−Smirnov test. Scores were examined as predictors of gut microbial groups, fecal SCFAs and blood biomarkers by regression analyses controlling by age and energy intake. These variables included *Akkermansia*, *Bacteroides-Prevotella-Porphyromonas*, *Bifidobacterium*, *Clostridia* cluster XIVa, *Lactobacillus* group, *Faecalibacterium prausnitzii*, acetic acid, propionic acid, butyric acid, glucose, triglycerides, Low density lipoprotein-high density lipoprotein (LDL-HDL) ratio, leptin, serum malondialdehyde (MDA), (total antioxidant capacity) TAC, C-Reactive protein (CRP), TGF-β, IL-10, IL-17, IL-8, IL-12, TNF-α, Phagocytosis granulocytes (%), Phagocytosis granulocytes and monocytes (%) and NK cell activity. When the distribution of variables was skewed (CRP, TGF-β, IL-10, IL-17, IL-8, IL-12, TNF-α) the values were converted to their natural logarithm. Association of microbial groups with food groups, macronutrients and micronutrients were evaluated through linear regression analyses adjusting by age and energy. The resulting data were plotted in a heatmap using the “pheatmat” function of the R program (version 3.5.1 for Windows). A Pearson correlation test was performed to elucidate closeness among dietary indices. This information was introduced in the program R, using the package “pheatmap,” to clusterize and plotting indices based on Euclidean distances. To test the association among dietary indices and previously reported health-beneficial dietary compounds, linear regression analyses were performed and plotted as forehead mentioned. To indicate statistical significance in the interpretation of results, the probability value of 0.05 was used.

## 3. Results

The average score on dietary indices according to general characteristics and socio-economic status, lifestyle and health-related factors of the studied sample is presented in Table 1. Among all the variables examined, only significant differences were observed for the age and these were found in all the studied dietary indices. Subjects over 65 years presented worse dietary scores than those in the group of 50–65 years. At the time of interpreting the results it should be taken into account that unlike the rest of the indices studied, in DII and EDII a higher score is associated with a more pro-inflammatory diet. Both DII and DQI-I showed better scores in people with energy intake higher than 1994.8 kcal. EDII score was found to be higher (worse dietary quality) as BMI increases. A lower score in the AHEI, DQI-I and Mediterranean dietary indices (rMED, MMDS), associated with worse dietary quality, was found in those subjects with bad self-health perception. Therefore, age and energy intake have been introduced in further analyses carried out as a covariate.

A general description of the variables that are subsequently analyzed in the study according to age groups is shown in Table 2. The levels of the bacterial groups analyzed were in the range of those previously reported in similar populations and demonstrated the large inter-individual variability present in the human adult fecal microbiota. Significant differences were observed in most of the microbiological parameters analyzed according to age. Subjects over 65 years of age presented lower fecal levels of *Bacteroides-Prevotella-Porphyromonas* group, *Clostridia* cluster *XIVa* and *Faecalibacterium,* as well as all the short chain fatty acids determined. Blood parameters are within the normal physiological ranges and were similar between the groups evaluated except for MDA, IL-8, IL-12 and TNF-α, whose concentration is higher in subjects over 65 years of age.

**Figure 1:**
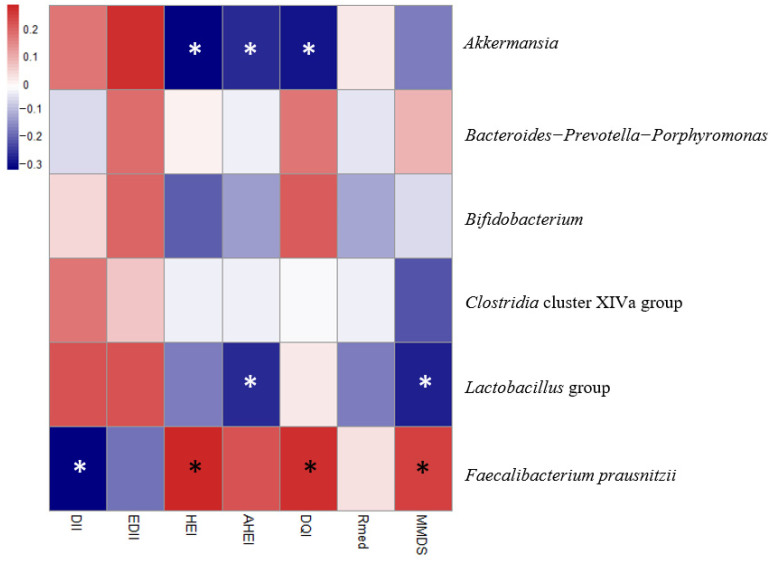
Heatmap defined by Pearson’s correlations between dietary indices scores and intestinal microbial groups. Blue and red colors represent negative and positive associations, respectively. The color intensity is proportional to the degree of association. Asterisks indicate correlation significance: * *p* ≤ 0.05.

In order to analyze possible linear relationships among the different dietary indices scores and fecal microbial groups, a linear regression analysis was conducted and a heatmap was plotted (Figure 1). Lower scores on indices related to diet quality (HEI, AHEI, DQI-I), suggestive of an unhealthier diet, were associated with increased *Akkermansia* levels. While DII, HEI, DQI-I and MMDS have shown potential as predictors of *F. prausnitzii*, which showed higher levels in those individuals with healthier diets, only AHEI and MMDS were negatively associated with *Lactobacillus* levels. These results were further examined including other health-related parameters in the analysis (Table 3). In relation to the production of SCFAs, higher scores in HEI, AHEI and MMDS indices were positively associated with the formation of acetic, propionic and butyric acids. Furthermore, as expected, inflammatory indices (DII, EDII) were the best determinants of lipoperoxidation blood levels, while Mediterranean ones were the best identifiers of serum IL-8 concentrations.

To deepen into how dietary components may modulate gut microbiota and SCFAs, linear regressions models adjusting by age and energy intake were applied and β-coefficient values were plotted in the heatmap of Supplementary Figure S1. Sauces and dips seemed to be the most significant food group for determining *Clostridia* cluster XIVa and *Lactobacillus* groups. However, the consumption levels of this food group were very low in our sample (data not shown) so that this association should be considered with caution. *F. prausnitzii* showed a positive association with fruits, legumes and with fiber, whereas a negative association was found for saturated fats. SCFAs appeared to be mostly related to oils and fats, seafood, total polyphenols and saturated fats.

**Figure 2:**
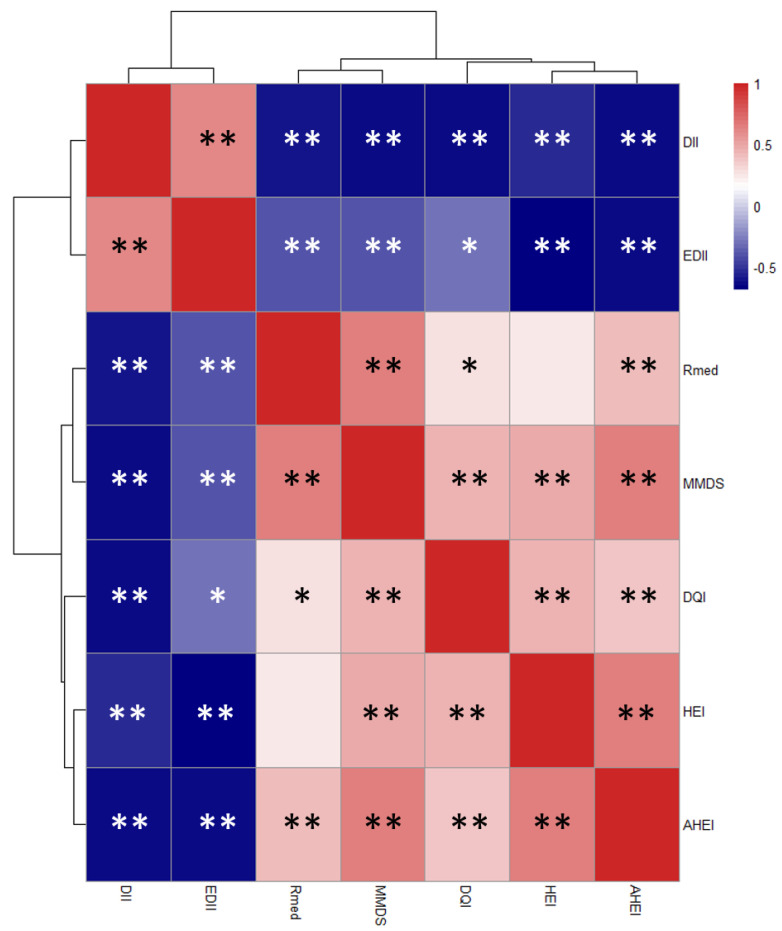
Heatmap defined by Pearson’s correlations between dietary indices scores. Blue and red colors represent negative and positive association, respectively. The color intensity is proportional to the degree of association between indices. Asterisks indicate correlation significance: * *p* ≤ 0.05; ** *p* ≤ 0.01. Due to the scale of the Dietary inflammatory index (DII) and the Empirical Dietary Inflammatory Index (EDII), they show an inverse relationship with the rest of the indices.

Heatmap showing Pearson´s correlation and clusterization among the different dietary indices is presented in Figure 2. Here, inflammatory indices revealed to be closer between them, forming the first cluster while the others integrate the second cluster. At the same time, HEI and AHEI grouped more closely with DQI-I and then with rMED and MMDS.

**Figure 3:**
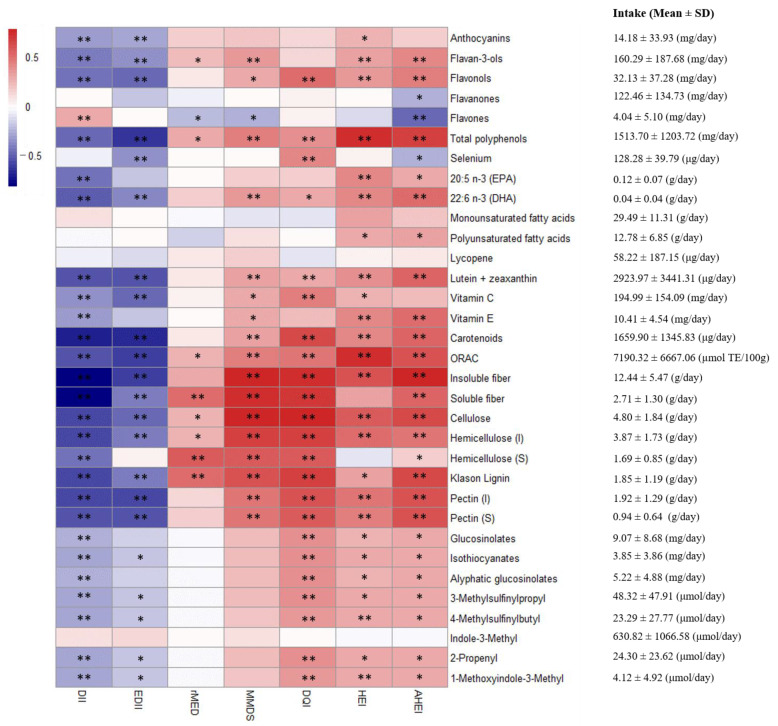
Heatmap showing β-coefficient values resulting from univariate linear regressions adjusting by age and energy among six different dietary indexes and health-related compounds. Rows include compounds with previously reported beneficial effect as (poly)phenols, fatty acids, carotenoids, vitamins, fiber, glucosinolates and isothiocyanates. Blue and red colors denote negative and positive association, respectively. Asterisks indicate the significance of the association degree * *p* ≤ 0.05; ** *p* ≤ 0.01.

To elucidate the relationship of dietary indices scores with the consumption of dietary compounds with reported anti-inflammatory and health-protective effects in the literature, a new linear regression analysis was performed adjusting by age and energy and a heatmap was plotted with the results (Figure 3). DII showed more negative correlation with vitamins, fiber, glucosinolates and isothiocyanates than EDII. All indices were associated with total polyphenols and ORAC. Except for rMED, indices had a significant correlation with flavonols, DHA, lutein + zeaxanthin, carotenoids, insoluble fiber and pectin. Glucosinolates and isothiocyanates did not show a significant association with the Mediterranean indices. In general, splitting indices into inflammatory, Mediterranean and diet quality ones, heatmap revealed similar patterns of association for the indices within each group. With the exception of flavones, all evaluated compounds revealed a negative association with inflammatory indices but positive with the Mediterranean and diet quality indices.

## 4. Discussion

In recent years, dietary indices have been a major step towards addressing the diet-health binomial from a global perspective. While tailored to different populations and constructed for specific purposes, a high degree of similarity can be observed among some of them. To our knowledge, this is the first study comparing the usefulness of dietary indices as predictors of human gut microbiota, the production of fecal SCFAs and the concentration in blood of different parameters related to the immune and inflammatory status, in a sample of middle-aged and elderly subjects without diagnosed pathology. As it has been previously proposed by other authors, for some of the indices included in the study, our data showed the existence of differences according to age. Furthermore, changes in the levels of some bacterial groups such as *Akkermansia* [44] or butyrate-producing bacteria, mainly *F*. *prausnitzii* [45,46], have been reported in aged individuals. Therefore, the factor “age” has been introduced in the models as a covariate in order to improve the interpretation of results.

Limitations in the use of dietary indices need to be also taken into account. DII encompasses a total of 45 components, of which 35 were evaluated in this sample. The effects of lacking 10 components in the scoring system may be attenuated by the own nature of the index and the fact of having a sample that does not show extreme values of consumption for any component. Moreover, nutritional supplements, weighted in other versions of EDII to compute the final score, were not included here. Some dietary indices such as EDII, HEI or AHEI were developed in the context of almost fully “westernized” societies, which could drive to an underrating or overrating in diets of populations with mixed diets (Mediterranean, African, etc.). Indeed, in our sample, the component “Trans Fat” that is evaluated in the AHEI, showed very low values while in typical EEUU diet is probably highly present. This may entail a loss of power in the accuracy of the prediction of our scores. Furthermore, DQI-I incorporates dietary variety, adequacy and moderation as a quality criterion. Although all these parameters have been included, the accuracy of FFQ to provide an accurate measurement of variety may be one of the limitations of this work. One of the main difficulties arises in the capacity of the indices to classify the subjects under study. In this sense, the scores from dietary quality indices showed low variability inter subject, with almost the whole sample obtaining scores indicative of poor or average diet quality for DQI-I, HEI and AHEI. Thus, we propose these indices as the worse in differentiating the poor-quality Mediterranean-style diet from a middle-age-elderly sample. On the other hand, indices related to adherence to a Mediterranean-style dietary pattern have presented a wide range of scores in the sample, ranging from 2–12 points and 1–7 for rMED and MMDS, respectively.

Based on the data obtained, the identification of the best tool to predict the composition and metabolic activity of the gut microbiota as a function of diet, is a difficult task. We considered that in the present study, the HEI, AHEI and DQI-I resulted likely inappropriate as predictor variables of differences between different microbiota as poor diets, that are different but score similarly, may mask trends associated to specific dietary constituents [47]. Interestingly, we found decreased levels of the mucin degrading *Akkermansia* in better scoring individuals compared to increased levels of *Akkermansia* in the worse ones, mainly influenced by vegetable consumption (data not shown). When vegetables are included at significant levels in diet, fiber consumption increases, which could promote the rise of some fiber-degrading species at the expense of other microorganisms such *Akkermansia* [48]. This could contrast with the fact that the presence of *Akkermansia* has been associated with healthy intestine and its abundance has been inversely correlated to several disease states [49,50,51,52,53,54]. We propose Mediterranean indices and more precisely MMDS, as the most accurate and best predictor in our population sample. Probably, socio-geographical reasons do Mediterranean indices the most suitable ones to measure the quality of diet in the sample and therefore, to predict microbiological and immunological variables. Also, some dietary indices related with inflammation (DII), quality of diet (HEI and DQI-I) and adherence to the Mediterranean-diet (MMDS) seem to be predictors of *F. prausnitzii* fecal levels, which were higher in individuals with healthier diets. *F. prausnitzii*, a member of the commensal microbiota, has been related with intestinal health and gut homeostasis [55]. Several studies highlight the anti-inflammatory properties of *F. prausnitzii* and its ability for upregulating T cell production and reducing IL-8 levels by blocking the NF-kB activation [56,57]. *F. prausnitzii* is a member of *Clostridium* cluster IV, one of the main producers of butyrate in the human colon [58] during fermentation of nondigestible polysaccharides such as dietary fiber. Butyrate plays several pleiotropic effects on host physiology and enhances the protection against pathogen invasion [59,60]. Remarkably, *Faecalibacterium* was found to be at high abundance in an Irish elderly sample [61] whereas some studies showed decreased levels of *F. prausnitzii* in centenarians as compared with younger adults [62]. Further studies are needed to determine the role of these bacteria in the intestinal microbiota of elderly populations.

The indices AHEI and MMDS (related to quality of diet and adherence to Mediterranean diet, respectively) were negatively associated with intestinal *Lactobacillus* levels. Increased *Lactobacillus* levels have been correlated with a higher PUFA/SFA ratio intake, probably mediated by changes in bile acid secretion and composition [23,63,64]. Extra virgin olive oil is an important component of Mediterranean diet and is a source of unsaturated fatty acids that can be metabolized by some intestinal *Lactobacillus* species [65,66,67]. Dietary indices used in the present work add from 0 to different positive numerical values to the formula relating dietary fats, as depending on the type and amount of fat consumed (calculated as a percentage of total energy intake). Therefore, as higher scores in dietary indices are generally accompanied in the general population by lower consumption of all type of fats (correlation values of “Lipids” with AHEI and MMDS of −0.154 and −0.149, respectively), this could provide a rationale to the inverse association found by us between scores for AHEI and MMDS and fecal levels of *Lactobacillus*. In this regard, we recently reported increased levels of the *Lactobacillus* group in Spanish adults displaying altered profiles of serum free fatty acids, which were accompanied by subclinical metabolic alterations [68].

Regarding the fecal SCFAs evaluated in our sample, acetic and propionic acids, correlated positively with heathier dietary scores for most of the indices. Microorganisms colonizing the gastrointestinal tract can participate in beneficial interactions within the intestinal ecological niche, as modulated by external factors such as diet. This is the case of the increase of the intestinal butyrate production by cross-feeding mechanisms. In cross-feeding, fiber-degrading bacteria can produce acetate as an end-product of fermentation, which is then metabolized by other members of the intestinal microbiota, as those belonging to *Clostridia* clusters XIVa and IV, to produce butyrate as an end-product of fermentation [51]. Both, bacteria and SCFAs contribute to cell expansion, immunosuppressive functions and overall intestinal homeostasis. Therefore, better dietary scores could be related with an enhanced SCFAs production in the gut [23].

Inflammatory indices have been identified as good predictors of inflammation variables such as CRP, IL-6 and TNF-α receptor 2 [12,13,14,69,70]. MDA is considered an oxidative stress biomarker that reflects levels of lipoperoxidation in blood. However, to the best of our knowledge, there are no previous reports relating dietary inflammatory indices and MDA. The lower detection of IL-8 in those individuals in the present work showing better scores on the indices related with the adherence to the Mediterranean diet may be partially explained by the increased levels of the anti-inflammatory bacterium *F. prausnitzii* [71] in such individuals. This bacterium was able to block the production of the inflammatory interleukin IL-8 in Crohn disease patients and in a murine colitis model [72].

## 5. Conclusions

The associations found among intestinal bacterial groups, SCFAs, blood biomarkers and dietary indices are indirectly reflecting how these variables are influenced by the specific components or food groups scoring in each index. When trying to discern differences among indices by clustering them, they are split in 3 main classes: inflammatory, diet quality and adherence to Mediterranean diet. The methodologies followed to construct them, the population they target and the scoring criteria define their nature and the way they correlated with others. The extension of the usefulness of dietary indices may shed some light into how to modulate gut microbiota focusing on dietary patterns.

## Figures and Tables

**Table 1:** Dietary indices score according to different characteristics of the sample.

Variable	n	DII	EDII	HEI	AHEI	DQI-I	rMED	MMDS
Mean (IQR)		−0.35 (1.07–−2.25)	0.67 (1.17–0.19)	58.16 (65.68–49.95)	60.99 (67.97–55.08)	49.03 (55–44)	7.16 (9–6)	3.37 (4–2)
Gender								
Male	20	−1.04 ± 2.27 _a_	0.64 ± 0.73 _a_	55.92 ± 11.47 _a_	63.48 ± 8.51 _a_	47.15 ± 9.53 _a_	7.80 ± 2.48 _a_	3,65 ± 1,31 _a_
Female	53	−0.10 ± 2.54 _a_	0.68 ± 0.78 _a_	59.00 ± 10.00 _a_	60.10 ± 10.52 _a_	49.74 ± 7.04 _a_	6.92 ± 2.28 _a_	3.26 ± 1.46 _a_
Age (years)								
50–65	33	−1.92 ± 2.05 _a_	0.24 ± 0.62 _a_	62.63 ± 9.01 _a_	64.07 ± 8.94_ a_	51.97 ± 0.83_ a_	8.39 ± 2.14 _a_	3.67 ± 1.29 _a_
>65	40	0.98 ± 2.02 _b_	1.02 ± 0.69 _b_	54.46 ± 10.16 _b_	58.39 ± 10.37 _b_	46.60 ± 5.96 _b_	6.15 ± 2.03 _b_	3.13 ± 1.49_ a_
Energy intake (kcal/day)								
≤1538.9	25	0.01 ± 2.3_ a,b_	0.41 ± 0.67_ a_	58.95 ± 11.43_ a_	63.49 ± 8.21_ a_	46.36 ± 7.76_ a_	7.08 ± 2.33_ a_	3.52 ± 1.66_ a_
1539–1994.8	24	0.40 ± 2.19_ a_	0.77 ± 0.62_ a_	57.22 ± 9.42_ a_	60.17 ± 9.80_ a_	47.46 ± 7.17_ a_	6.79 ± 1.86_ a_	3 ± 1.02_ a_
>1994.9	24	−1.47 ± 2.65_ b_	0.84 ± 0.92_ a_	58.27 ± 10.67_ a_	59.32 ± 11.84_ a_	53.38 ± 6.88_ b_	7.63 ± 2.76_ a_	3.58 ± 1.47_ a_
BMI (kg/m^2^)								
<25	19	−0.99 ± 2.77_ a_	0.37 ± 0.72_ a_	10.02 ± 19_ a_	62.74 ± 9.90_ a_	48.95 ± 9.20_ a_	7.79 ± 2.32_ a_	3.68 ± 1.60_ a_
25.0–29.9	37	−0.39 ± 2.37_ a_	0.62 ± 0.77_ a.b_	10.10 ± 37_ a_	61.83 ± 10.16_ a_	48.43 ± 7.65_ a_	7.11 ± 2.50_ a_	3.22 ± 1.38_ a_
≥30	17	0.44 ± 2.33_ a_	1.10 ± 0.62_ b_	10.35 ± 17_ a_	57.28 ± 9.77_ a_	50.41 ± 6.75_ a_	6.59 ± 1.97_ a_	3.35 ± 1.32_ a_
Depositions (times/week)								
≤3	8	0.51 ± 2.12 _a_	0.85 ± 0.58_ a_	53.09 ± 12.71_ a_	60.29 ± 6.12_ a_	47.50 ± 7.96_ a_	7.00 ± 2.51_ a_	3.00 ± 1.60_ a_
>3–7	57	−0.11 ± 2.48 _a_	0.71 ± 0.76_ a_	58.40 ± 10.02_ a_	60.74 ± 10.44_ a_	49.19 ± 7.89_ a_	6.93 ± 2.34_ a_	3.35 ± 1.38_ a_
>7	8	−2.82 ± 1.28_ a_	0.16 ± 0.85_ b_	61.51 ± 10.59_ a_	63.44 ± 10.92_ a_	49.38 ± 8.07_ a_	9.00 ± 1.51_ a_	3.88 ± 1.55_ a_
Smoking status								
Never	9	−2.27 ± 1.74_ a_	0.11 ± 0.51_ a_	67.14 ± 8.34_ a_	64.66 ± 7.90_ a_	49.22 ± 10.94_ a_	8.78 ± 2.54_ a_	4.0 ± 1.32 _a_
Current	17	−0.40 ± 2.55_ a_	0.63 ± 0.67_ a_	60.79 ± 9.12_ a_	61.14 ± 8.21_ a_	50.35 ± 6.72_ a_	7.53 ± 2.24_ a_	3.65 ± 1.37_ a_
Former	16	−1.11 ± 2.04_ a_	0.38 ± 0.59_ a_	60.17 ± 10.28_ a_	65.93 ± 7.43_ a_	49.81 ± 8.23_ a_	8.25 ± 1.77_ a_	3.75 ± 1.18_ a_
Educational level								
None or primary school	2	−2.46 ± 0.42_ a_	0.45 ± 0.49_ a_	66.19 ± 5.27_ a_	63.13 ± 7.38_ a_	57.00 ± 7.07_ a_	7.00 ± 1.41_ a_	3.50 ± 0.71_ a_
Technical school	5	−1.73 ± 1.80_ a_	−0.03 ± 0.34_ a_	63.81 ± 6.21_ a_	63.80 ± 1.99_ a_	51.60 ± 6.35_ a_	9.60 ± 1.67_ a_	3.20 ± 1.10_ a_
Secondary school	7	−1.40 ± 2.86_ a_	0.37 ± 0.65_ a_	59.20 ± 8.96_ a_	60.29 ± 8.83_ a_	54.00 ± 8.06_ a_	7.71 ± 2.75_ a_	3.29 ± 1.25_ a_
University degree	13	−2.00 ± 1.61_ a_	0.19 ± 0.46_ a_	63.55 ± 9.67_ a_	66.63 ± 7.77_ a_	48.85 ± 9.14_ a_	8.54 ± 1.90_ a_	4.00 ± 1.22_ a_
Mood feeling								
Really satisfied	42	−0.35 ± 2.61_ a_	0.70 ± 0.81_ a_	57.99 ± 10.07_ a_	60.63 ± 9.90_ a_	48.88 ± 8.16_ a_	7.24 ± 2.54_ a_	3.48 ± 1.45_ a_
Satisfied	21	−0.68 ± 2.28_ a_	0.57 ± 0.65_ a_	61.28 ± 10.14_ a_	63.27 ± 10.61_ a_	49.48 ± 7.61_ a_	7.76 ± 1.84_ a_	3.38 ± 1.40_ a_
Unsatisfied	8	0.96 ± 2.17_ a_	0.78 ± 0.58_ a_	51.65 ± 11.64_ a_	57.86 ± 7.67_ a_	46.75 ± 6.90_ a_	5.25 ± 1.98_ a_	2.63 ± 1.30_ a_
Self-health perception								
Excellent-good	42	−0.01 ± 2.20_ a_	0.75 ± 0.72_ a_	57.17 ± 9.85_ a_	59.24 ± 7.88_ a_	47.12 ± 7.15_ a_	6.95 ± 2.39_ a,b_	3.26 ± 1.31_ a,b_
Normal	18	−1.53 ± 2.54_ a_	0.51 ± 0.68_ a_	61.38 ± 101.39_ a_	66.44 ± 10.49_ b_	52.28 ± 7.89_ a_	8.33 ± 2.22_ a_	4.00 ± 1.57_ a_
Bad	12	0.56 ± 2.76_ a_	0.75 ± 0.95_ a_	56.43 ± 11.15_ a_	58.14 ± 13.62_ a,b_	49.92 ± 8.40_ a_	6.08 ± 1.88_ b_	2.67 ± 1.23_ b_

First row values are the mean of the corresponding index in the sample followed by the interquartile range (IQR = Q3 − Q1). The rest of values are shown as mean ± standard deviation (SD). For a certain variable showing two of more subgroups, only values in the same column with different subscript present a statistically significant difference (*p* ≤ 0.05). Mood feeling refer to volunteer’s awareness about the satisfaction with their life. Self-perception of health status refer to individual perception about health-quality degree. Inflammatory indices (DII, Dietary Inflammatory Index. EDII, Empirical Dietary Inflammatory Index): negative values favor non-inflammatory states and positives values enhance inflammation. Dietary Quality indices (HEI, Healthy Eating Index. AHEI, Alternative Healthy Eating Index. DQI-I, Diet Quality Index-International): higher scores are reflecting consumption values similar to those recommended in dietary guidelines. Mediterranean Dietary indices (rMED, Relative Mediterranean Diet Score. MMDS, Modified Mediterranean Diet Score): higher values showing a higher degree of adherence to the Mediterranean diet.

**Table 2:** Microbial levels, short chain fatty acid (SCFA) concentration and blood biomarkers according to age groups.

Variable	Age Groups	
	G1 (≤65) (*n* = 33)	G2 (>65) (*n* = 40)
*Akkermansia* (Log_10_ n° cells/gram feces)	6.43 ± 1.88 _a_	6.99 ± 1.77 _a_
*Bacteroides-Prevotella-Porphyromonas* (Log_10_ n° cells/gram feces)	9.32 ± 0.82 _a_	8.79 ± 0.69 _b_
*Bifidobacterium* (Log_10_ n° cells/gram feces)	7.93 ± 1.53 _a_	7.55 ± 1.10 _a_
*Clostridia* cluster XIVa (Log_10_ n° cells/gram feces)	7.57 ± 1.49 _a_	6.45 ± 1.54 _b_
*Lactobacillus* group (Log_10_ n° cells/gram feces)	5.91 ± 1.26 _a_	6.97 ± 1.83 _b_
*Faecalibacterium prausnitzii* (Log_10_ n° cells/gram feces)	7.07 ± 0.76 _a_	6.42 ± 1.31 _b_
Acetic acid (mM)	29.81 ± 9.25 _a_	23.18 ± 14.45 _b_
Propionic acid (mM)	12.94 ± 5.43 _a_	9.50 ± 7.46 _b_
Butyric acid (mM)	11.76 ± 9.39 _a_	8.44 ± 7.94 _a_
Glucose (mg/dL)	97.76 ± 12.99 _a_	106.78 ± 33.85 _a_
Triglycerides (mg/dL)	118.82 ± 54.23 _a_	121.75 ± 48.41 _a_
Cholesterol (mg/dL)	233.18 ± 39.06 _a_	203.39 ± 37.48 _b_
LDL-HDL ratio	2.76 ± 0.78 _a_	2.72 ± 0.81 _a_
Leptin (ng/mL)	9.62 ± 5.72 _a_	12.01 ± 7.87 _a_
Serum MDA (μM)	2.01 ± 0.53 _a_	2.60 ± 0.49 _b_
Antioxidant capacity (mM)	0.34 ± 0.09 _a_	0.35 ± 0.09 _a_
CRP (mg/L)	1.28 ± 1.22 _a_	1.19 ± 1.03 _a_
TGF-β (ng/mL)	4.44 ± 2.71 _a_	6.25 ± 5.70 _a_
IL-10 (pg/mL)	0.14 ± 0.78 _a_	0.80 ± 3.70 _a_
IL-17 (pg/mL)	1.42 ± 3.10 _a_	2.28 ± 11.57 _a_
IL-8 (pg/mL)	7.09 ± 6.01 _a_	20.80 ± 9.91 _b_
IL-12 (pg/mL)	0.21 ± 1.21 _a_	3.54 ± 8.92 _b_
TNF-α (pg/mL)	0.23 ± 1.23 _a_	4.82 ± 7.94 _b_
Phagocytosis granulocytes (%)	72.23 ± 22.13 _a_	86.37 ± 18.53 _a_
Phagocytosis granulocytes and monocytes (%)	71.39 ± 21.32 _a_	82.35 ± 17.49 _a_
NK cell activity (%)	53.09 ± 11.25 _a_	52.70 ± 18.19 _a_

Values are shown as mean ± standard deviation (SD). Values in the same row showing different subscripts present a statistically significant difference (*p* ≤ 0.05).

**Table 3:** Results obtained from regression analyses to identify dietary indices as predictors of gut microbiota levels (log_10_ n°. cells per gram of feces), fecal short chain fatty acids (mM) and blood biomarkers.

	Dependent Variable	Independent Variable	*R* ^2^	β	*p*
Model 1. Fecal microbiota groups *Akkermansia, Bacteroides-Prevotella-Porphyromonas*, *Bifidobacterium*, *Clostridia* cluster XIVa, *Faecalibacterium prausnitzii*, *Lactobacillus* group	*Akkermansia*	HEI	0.080	−0.307	0.026
		AHEI	0.059	−0.256	0.050
		DQI-I	0.072	−0.285	0.038
	*Faecalibacterium prausnitzii*	DII	0.124	−0.312	0.030
		HEI	0.128	0.284	0.035
		DQI-I	0.122	0.265	0.047
		MMDS	0.123	0.240	0.044
	*Lactobacillus* group	AHEI	0.264	−0.256	0.027
		MMDS	0.283	−0.275	0.012
Model 2. Fecal short chain fatty acids Acetic acid, Propionic acid, Butyric acid	Acetic acid	DII	0.252	−0.425	0.003
		EDII	0.244	−0.369	0.013
		HEI	0.239	0.320	0.016
		AHEI	0.335	0.478	0.000
		MMDS	0.356	0.451	0.000
	Propionic acid	DII	0.198	−0.316	0.031
		HEI	0.246	0.348	0.009
		AHEI	0.303	0.441	0.000
		MMDS	0.292	0.378	0.001
	Butyric acid	HEI	0.189	0.289	0.034
		AHEI	0.213	0.338	0.007
		MMDS	0.211	0.298	0.012
Model 3. Blood biomarkers Glucose, Triglycerides, LDL-HDL ratio, Leptin, Serum malondialdehyde (MDA), Antioxidant capacity, C-Reactive protein (CRP), Transforming growth factor-beta (TGF-β), IL-10, IL-17, IL-8, IL-12, TNF-α, % Phagocytosis granulocytes, % Phagocytosis granulocytes and monocytes, NK cell activity	MDA	DII	0.297	0.373	0.003
		EDII	0.318	0.408	0.002
	IL-8	rMED	0.443	−0.251	0.018
		MMDS	0.443	−0.221	0.017

Linear regression analyses are adjusted by age and energy. R^2^, coefficient of multiple determination; β, standardized regression coefficient for examined variable. *p* ≤ 0.05. Inflammatory indices (DII, Dietary Inflammatory Index. EDII, Empirical Dietary Inflammatory Index): negative values favor non-inflammatory states and positives values enhance inflammation. Dietary Quality indices (HEI, Healthy Eating Index. AHEI, Alternative Healthy Eating Index. DQI-I, Diet Quality Index-International): higher scores are reflecting consumption values similar to those recommended in dietary guidelines. Mediterranean Dietary indices (rMED, Relative Mediterranean Diet Score. MMDS, Modified Mediterranean Diet Score): higher values showing a higher degree of adherence to the Mediterranean diet.

## Supplementary Materials

The following are available online at https://www.mdpi.com/2072-6643/12/12/3828/s1, Figure S1: Heatmap showing β-coefficient values resulting from univariate linear regressions adjusting by age and energy among microbial groups, SCFAs and food groups and dietary compounds, Table S1: Characteristics of dietary indexes, Table S2: Primers and annealing temperatures used for the quantification of intestinal microbial groups by qPCR.
