# Supplementary material for: Comparison of Different Dietary Indices as Predictors of Inflammation, Oxidative Stress and Intestinal Microbiota in Middle-Aged and Elderly Subjects
Source: Nutrients. 2020 Dec 15;12(12):3828. doi: 10.3390/nu12123828 (PMC7765160; doi:10.3390/nu12123828)
Supplement: Supplementary file 1 [file nutrients-12-03828-s001.pdf]

Table S1. Characteristics of dietary indexes.

Index	Nº components	Components	Total value range (From-to)	Group classification [1]	Reference
Dietary Inflammatory Index (DII)	45 (35) [2]	Ethanol, Vitamin B12, Vitamin B6, β-carotene, Caffeine, Carbohydrates, Cholesterol, Energy, Total fat, Fibre, Folic acid, Garlic, Fe, Mg, MUFA, Niacin, *n*-3 fatty acids, *n*-6 fatty acids, Onion, Protein, PUFA, Riboflavin, Saturated fat, Se, Thiamin, Trans fat, Vitamin A, Vitamin C, Vitamin D, Vitamin E, Flavan-ol, Flavones, Flavonols, Flavonones, Anthocyanidins	−8.87 - 7.98	-8.87 – 0 = Anti-inflammatory<br>0.01 – 7.98 = Pro-inflammatory	[7]
Empirical Dietary Inflammatory Index (EDII)	18	Processed meat, Red meat, Organ meat, Other fish, Other vegetables, Refined grains, High-energy beverages, Low-energy beverages, Tomatoes, Beer, Wine, Tea, Coffee, Dark yellow vegetables, Leafy green vegetables, Snacks, Fruit juice, Pizza	Undefined	X ≤ 0 = Anti-inflammatory<br>X ≥ 0 = Pro-inflammatory	[8]
Healthy Eating Index - 2015 (HEI)	13	Total fruits, Whole fruits, Total vegetables, Greens and beans, Whole grains, Dairy, Total protein foods, Seafood and plant proteins, Fatty acids, Refined grains, Sodium, Added sugars, Saturated fats	0 - 100	0 – 59 = F<br>60 – 69 = D<br>70 – 79 = C<br>80 – 89 = B<br>90 – 100 = A	[15]
Alternative Healthy Eating Index – 2010 (AHEI)	11	Vegetables, Fruits, Whole grains, Sugar-sweetened beverages and fruit juice, Nuts and legumes, Red/processed meat, Trans fat, Long-chain	0 - 110	Undefined	[10]

		(n-3) fats (EPA + DHA), PUFA, Sodium, Alcohol			
Diet quality Index-International b adapted (DQI-I)	18	Overall food group variety, Within-group variety for protein sources, Vegetable group, Fruit group, Grain group, Fibre, Protein, Iron, Calcium, Vitamin C, Total Fat, Saturated Fat, Cholesterol, Sodium, "Empty calorie foods", Macronutrient ratio, Fatty acid ratio	0 - 100	0 ≤ 45 = Poor quality diet<br><br>> 55 - 60 = Medium quality diet<br><br>> 75 = High quality diet	[36]
Relative Mediterranean Diet (rMED)	9	Fruits, Vegetables, Legumes, Fish, Olive oil, Cereals, Total meat, Dairy products, Ethanol	0 - 18	0 – 6 = Low adherence<br><br>7 – 10 = Medium adherence<br><br>11 – 18 = High adherence	[37]
Modified Mediterranean Diet Score (MMDS)	9	Vegetables, Legumes, Fruits, Cereals, Fish, Meat, Dairy products, Ethanol, MUFA+PUFA/SFA	0 - 9	0 – 3 = Low adherence<br><br>4 – 5 = Medium adherence<br><br>6 – 9 = High adherence	[38]

* DII accounts for 45 items in total, of which 35 were evaluated in this study ** Dietary scores classification into groups may be useful to have way of interpreting them. MUFA, monounsaturated fatty acid; PUFA, polyunsaturated fatty acid; SFA, saturated fatty acid.

Table S2. Primers and annealing temperatures used for the quantification of intestinal microbial groups by qPCR.

Microbial Group	Primer sequence (5´-3´)	Tm. (ºC)
Akkermansia	F: CAGCACGTGAAGGTGGGGAC<br>R: CCTTGCGGTTGGCTTCAGAT	60
Bacteroides-Prevotella-Porphyromonas	F: GAGAGGAAGGTCCCCCAC<br>R: CGCKACTTGGCTGGTTCAG	60
Bifidobacterium	F: GATTCTGGCTCAGGATGAACGC<br>R: CTGATAGGACGCGACCCCAT	60
Clostridia cluster *XIVa* group	F: CGGTACCTGACTAAGAAGC<br>R: AGTTTYATTCTTGCGAACG	55
Faecalibacterium prausnitzii	F: GGAGGAAGAAGGTCTTCGG<br>R: AATTCCGCCTACCTCTGCACT	60
Lactobacillus group	F: AGCAGTAGGGAATCTTCCA<br>R: CATGGAGTTCCACTGTCCTC	60

Adapted from Valdes et al. [42]

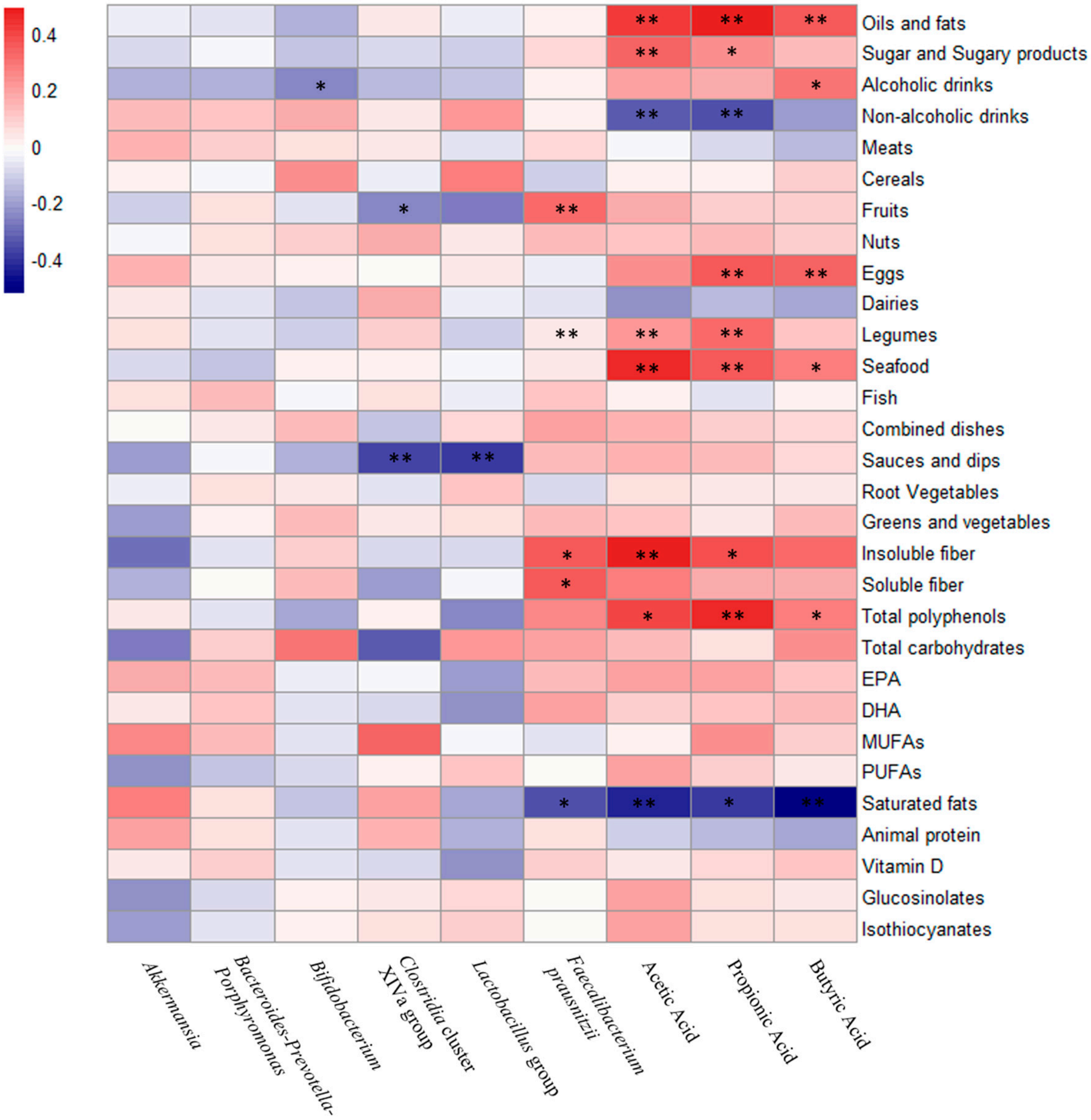

Figure S1. Heatmap showing β-coefficient values resulting from univariate linear regressions adjusting by age and energy among microbial groups, SCFAs and food groups and dietary compounds. Rows include 17 main food groups and others such as fiber, total polyphenols, total carbohydrates, fatty acids, animal protein, vitamin D and glucosinolates, all of them previously found by other authors to be related with the growth of a bacterial group at least. Blue and red colors denote negative and positive association, respectively. Asterisks indicate the statistical significance of the association degree * $p \leq 0.05$; ** $p \leq 0.01$.
